# The Flourishing Child: Study Protocol for an Acceptability and Feasibility Trial of a Digital Early Childhood Flourishing Intervention

**DOI:** 10.3390/children13050581

**Published:** 2026-04-22

**Authors:** Zenobia Talati, Jack Kennare, Natasha L. Bear, Lisa Y. Gibson, Robyn Power, Van Zyl Kruger, Desiree Silva, Susan L. Prescott, Jacqueline A. Davis

**Affiliations:** 1 The Kids Research Institute Australia, Perth 6009, Australia; zenobia.talati@thekids.org.au (Z.T.); jack.kennare@thekids.org.au (J.K.); natasha@bearstats.com.au (N.L.B.); lisa.gibson@thekids.org.au (L.Y.G.); vanzyl.kruger@datadivers.io (V.Z.K.); desiree.silva@thekids.org.au (D.S.); 2 School of Medicine, The University of Western Australia, Perth 6009, Australia; susan.prescott@thekids.org.au; 3 School of Population Health, Curtin University, Perth 6102, Australia; 4 School of Medical and Health Sciences, Edith Cown University, Perth 6027, Australia; 5 Joondalup Health Campus, Perth 6027, Australia; 6 Nova Institute for Health, Baltimore, MD 21231, USA; 7 Family and Community Medicine, University of Maryland, Baltimore, MD 21201, USA

**Keywords:** flourishing, wellbeing, early years, parenting, digital intervention, protocol

## Abstract

**Highlights:**

**What are the objectives of the study protocol?**

- To describe methods to test the acceptability and feasibility of a digital intervention promoting flourishing in early childhood.
- To describe methods to collect and analyse pilot data measuring changes to parental self-efficacy.

**What are the anticipated implications of the main finding?**

- Findings will inform a future trial testing the effectiveness of the Flourishing Intervention.
- We anticipate that the findings will support the acceptability and feasibility of the intervention.

**Abstract:**

Background: Globally, rates of children with physical and mental health problems are increasing. Health issues in early childhood often persist into adulthood, highlighting the need to ensure children are supported to flourish from the start of life. Objectives: This protocol describes methods used to test the acceptability and feasibility of a novel digital Flourishing Intervention (designed to empower parents and promote child wellbeing), comprising a Flourishing Check (a newly developed online questionnaire) and a Pathway Tool (an online directory of high-quality, evidence-based programmes and resources). Methods: Using a randomised feasibility trial, participants (N = 600 parents of children aged 0–5 years) will complete the Flourishing Check. The intervention group (n = 400) will access the Flourishing Check and Pathway Tool, whereas a waitlist control group (n = 200) will access the Flourishing Check only. Results: The primary aim is to assess the acceptability and feasibility of the intervention through a mixed-methods design incorporating quantitative data from pre- and post-intervention questionnaires and qualitative data from focus groups. This will be assessed using a traffic light system, which will inform if and how to proceed to a future effectiveness trial. Secondary aims are to assess changes in parent and child outcomes. Primary outcomes will be assessed using descriptive statistics and thematic analysis. Secondary outcomes will be analysed using mixed-effects regression models. Conclusions: We anticipate that the Flourishing Intervention will be feasible and acceptable to parents. This trial is registered with the Australian New Zealand Clinical Trials Registry (ACTRN12626000187347).

## 1. Introduction

Supporting childhood mental and physical health in the early years (0–5 years) has been identified as a global research priority [1,2,3,4,5,6,7]. Mental health issues that develop during early childhood often persist into adulthood, preventing children from realising their potential and increasing long-term burden on families, economies, and health systems [2,8]. Insufficient support for this age range is a missed opportunity, as this is when developmental plasticity is at its peak [9].

Understanding when and how to access early intervention can be challenging for parents and caregivers [10] (hereafter referred to as “parents”). The World Health Organization (WHO) recommends developmental screening during children’s visits to health services in the first five years of life [11] as a window of opportunity to initiate early intervention. However, in Australia, disparities in access reduce attendance, and there is little nationally representative data on the uptake of these services [12]. The proportion of eligible children attending their free universal two-year child health checks in metropolitan WA is only 30% [13]. Families are underutilising these screening opportunities and cite a lack of awareness of the programmes and services available to them [14]. Parents need accessible pathways to navigate healthcare systems [15] but frequently feel overwhelmed by information and disempowered [10].

### 1.1. The Value of Digital Health Interventions in the Early Years

Digital interventions for early childhood aim to improve access to credible and evidence-based supports, parent and child wellbeing, and provide targeted pathways to intervention and prevention [16]. They have the potential to improve child and parent outcomes and are more accessible and cost-effective than face-to-face interventions [16,17,18]. Online interventions have been shown to reduce child problem behaviour and improve parental mental health [19]. Furthermore, online parental support has been shown to be non-inferior to face-to-face support in improving child and parent mental health, and more satisfactory to parents [20,21,22]. However, further research is needed to improve parental adherence to these interventions [23,24] and improve long-term benefits [16]. Given the importance of intervening early (i.e., in early childhood) [9] and the cost-effectiveness and scalability of digital interventions, their development and evaluation represent a research priority. Importantly, digital interventions to improve early childhood must be co-designed with and by intended users to ensure resulting interventions address their needs [25]. Co-design refers to the active involvement of stakeholders (e.g., end-users, service providers) in the development and implementation of solutions to pre-defined problems [26]. Meaningful co-design is maintained throughout all project stages, emphasises respect and collaboration, and is essential to the design of acceptable and feasible solutions [26,27,28]. Improving health outcomes for young children therefore relies on empowering parents and caregivers.

### 1.2. Flourishing in Early Childhood

Flourishing is a state of positive functioning in all areas of life, characterised by the presence of strengths or protective factors and the absence of symptoms and disease [29,30,31]. It goes beyond quality of life and wellbeing to encompass additional individual factors, including happiness and relationships, and environmental factors such as strong communities [31,32]. Recent reviews have concluded that there is dissensus among conceptualisations of flourishing in early childhood (0–5 years), and there is a need to develop frameworks and measures addressing this gap [32]. To define and operationalize early childhood flourishing, the current authors engaged community stakeholders (e.g., parents, early childhood professionals, and children) to understand what it means to them [10]. Responses were collected through self-report questionnaires, focus groups, and interviews with children, and analysed using a mixed-methods approach. This resulted in a community-informed framework comprising five domains of early childhood flourishing: physical safety, love and family, positive lifestyle, physical and mental health, and fun and happiness.

Additionally, key barriers and enablers to supporting early childhood flourishing were identified. Key barriers included time scarcity, competing commitments, and the cost and difficulty of securing appointments. Enablers included family support (e.g., extended family and friends), safety (physical and online), good health and nutrition, and sleep. Parents voiced concerns about the volume of parenting information and resources, and the lack of clarity and consensus among them. These responses suggested parents need interventions that (1) help them understand their children’s strengths and support needs (i.e., where they are flourishing and where they may benefit from support), and (2) provide targeted pathways to evidence-based supports.

Subsequently, a scoping review (under review) was conducted by the authors to assess alignment of the community-informed framework with the early childhood flourishing literature. Searches were conducted across nine databases to identify existing frameworks, measures, domains, and indicators of early childhood flourishing. Judgements of alignment were based on consensus among researchers on which domains and indicators identified in the scoping review results were theoretically linked to those in the pre-existing framework. Curiosity, learning, and engagement were identified as a gap, resulting in a revised framework comprising six domains: safety and security, love and connection, a positive lifestyle, wellbeing, fun and play, and curiosity. This framework aligns with and extends on existing flourishing frameworks such as the National Survey of Children’s Health Child Flourishing Index.

Within this framework, flourishing is conceptualised through both determinants and indicators. Certain environmental determinants (e.g., safety and security, love and connection, positive lifestyle) must be present for children to demonstrate the external indicators of flourishing (e.g., wellbeing, fun and play, and curiosity). Early childhood flourishing is therefore holistic and comprehensive, and distinct from existing constructs which are narrower in scope (e.g., wellbeing, quality of life, developmental outcomes).

There are many existing interventions that aim to reduce child problem behaviour or improve parenting skills [17,19,24]. The intervention described in this protocol sits apart from those in that it aims to prevent problem behaviours and, crucially, enhance strengths. Furthermore, it was developed through rigorous co-design, ensuring it addresses the specific needs of its intended users [10].

### 1.3. Mechanisms of Change

Sanders and Mazzucchelli [33] present a conceptual model outlining mechanisms of change by which parenting interventions lead to improved child outcomes. The primary mechanism is changes in parenting behaviours and attitudes (e.g., increased self-regulation, more positive interactions, less coercive interactions, and appropriate expectations of children and self). Within this model, parenting changes are influenced by engagement, which is in turn influenced by parental concerns (e.g., perceived need for support), wellbeing (e.g., mental health, attachment), cognitions (e.g., perceived self-efficacy), cultural and social influences, motivational contexts (e.g., anticipated child benefits), and intervention features (e.g., accessibility, format, cost, timing).

**Figure 1:**
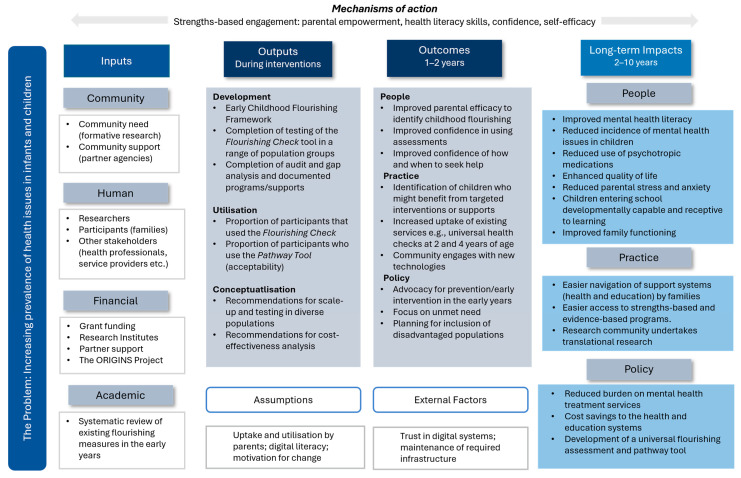
Logic model for the flourishing child.

A Logic Model incorporating a theory of change is a valuable tool for visually clarifying the intended pathways to expected outcomes and long-term change in complex health challenges [34]. For this study, the research team developed a logic model for enhancing flourishing in the early years to measure and evaluate project inputs, outcomes, and impacts (see Figure 1). Additionally, pre- to post-intervention changes to parental perceived self-efficacy, confidence, wellbeing, engagement, knowledge of when and how to seek help, and perceived need for support will be explored to better understand mechanisms of change.

### 1.4. The Flourishing Intervention

In this protocol, we describe the Flourishing Intervention, designed to promote child flourishing by improving parents’ awareness of their children’s strengths and support needs, and their knowledge of when to seek support. This digital health intervention comprises a Flourishing Check and a Pathway Tool, conceptualised by the research team with the aim of providing parents with both knowledge of and access to timely supports. The design and testing of the Flourishing Intervention are nested within a longitudinal cohort study, ORIGINS, based in Perth, Western Australia. ORIGINS [35] is a collaboration between The Kids Research Institute Australia and Joondalup Health Campus, and is one of the most comprehensive studies of pregnant women and their families in Australia.

### 1.5. The Flourishing Check

The Flourishing Check is a new questionnaire designed to enable parents to check in on their child’s level of flourishing and signpost domains where they may benefit from support. The reliability of children below five years of age to self-report is limited [36]. Therefore, this assessment relies on parent-proxy reports. It is underpinned by the early childhood flourishing framework described above, uses items uncovered through the scoping review (under review) into early childhood flourishing measures, and is also underpinned by The Nest [37] (an Australian framework which outlines population-level indicators of child and adolescent wellbeing). The Flourishing Check is available in two versions assessing flourishing in distinct developmental stages (0–2 years and 3–5 years). Assessment development followed best-practice guidelines [38], starting with a large item pool and adjusting/narrowing down the items using the Delphi method of consultation with experts. The questionnaire will be checked for test–retest reliability and validated against the Paediatric Quality of Life questionnaire (PedsQL) and the Ages and Stages Questionnaire–Socio-Emotional scale (ASQ-SE). A manuscript detailing the development and psychometric properties of the Flourishing Check is currently in preparation.

### 1.6. The Pathway Tool

**Figure 2:**
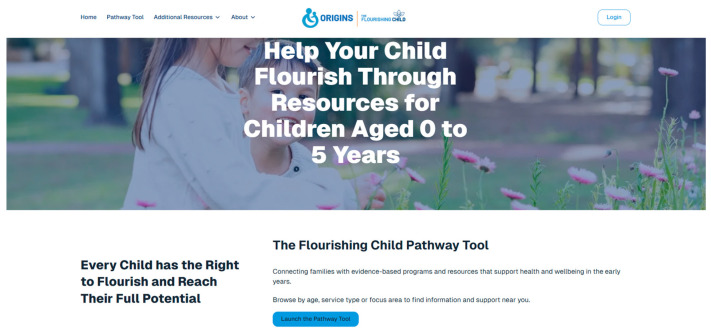
Screenshot of the Pathway Tool homepage.

The Pathway Tool is a digital information hub through which parents can find high-quality, evidence-based programmes and resources that support early childhood flourishing. Based on their responses to the Flourishing Check, the Pathway Tool website will signpost parents to specific domains of flourishing where their child may benefit from support and direct them to relevant, evidence-based programmes and resources. Figure 2 shows a screenshot of the Pathway Tool homepage. The Pathway Tool was developed by the project team in an iterative process involving a systematic search for eligible services, independent reviewers screening search results, and the application of a comprehensive quality assessment.

The Pathway Tool was primarily designed to be applicable to residents of the Joondalup and Wanneroo regions of Perth, Western Australia, as this is the location of the ORIGINS cohort [35]. Therefore, in-person programmes included in the tool will be most accessible to residents of this region. However, the tool contains resources and online content applicable to residents beyond this area (i.e., across broader Perth metropolitan regions). See Section S1 of the Supplementary Materials for further details on the development of the Pathway Tool.

### 1.7. Governance

This intervention was co-designed with stakeholders’ input at all project stages. Two groups of stakeholders have collaborated in its development and will have ongoing involvement in future project phases: (a) a Consumer and Community Advisory Committee comprising parents, early childhood educators, and community representatives, who ensured the intervention is relevant, culturally appropriate, and accessible, and (b) a Steering Committee including representatives from partner organisations representing the Australian early childhood sector, the local community, the interests of multicultural families and fathers, along with experts in digital design. Collectively, these committees have provided guidance in the form of lived experience [39], expertise in early childhood development, expertise in digital tool development, and diverse cultural perspectives. This process has resulted in an intervention that is informed by relevant knowledge and centred on the needs and priorities of intended end-users. For example, it was at the advice of these groups that the name of the Flourishing Check was changed from the original title of Flourishing Assessment (which was perceived as having a clinical/diagnostic undertone that did not align with the purpose of the questionnaire). This study will be coordinated at The Kids Research Institute Australia, with the corresponding author overseeing all aspects of trial conduct, including recruitment, consent, intervention delivery, and data collection. Data management, including data entry, storage, and quality checks, will be conducted by the research team under the supervision of the corresponding author. Given the low-risk, short-duration, single-site nature of the study, no separate endpoint adjudication committee or independent data management team has been established.

### 1.8. Aims

The primary aim of this study is to test the acceptability and feasibility of the Flourishing Intervention and explore any areas for modification required. Secondary aims are to collect pilot data to explore post-intervention changes in parent and child outcomes, including (i) perceived self-efficacy in recognising and supporting flourishing in early childhood, (ii) perceived knowledge of how and when to seek support, (iii) engagement with and participation in supports relevant to flourishing in the early years, (iv) parent wellbeing, and (v) paediatric quality of life. It is hypothesised that the intervention components will be deemed feasible and acceptable to the level where this pilot can proceed to a full trial (either with or without modifications).

## 2. Materials and Methods

### 2.1. Study Design

A randomised feasibility trial will be used to test the feasibility and acceptability of a new flourishing intervention and explore any areas for modification required (primary aim). A secondary aim will be to explore whether the intervention group shows any improvements in parent outcomes relative to controls after engaging with the Pathway Tool. This protocol is reported in accordance with SPIRIT guidelines and informed by the CONSORT extension for pilot and feasibility trials (see Section S2 of the Supplementary Materials) [40].

Participants will complete a screening form to determine eligibility and then be randomly allocated to either the intervention group (which will gain access the Pathway Tool immediately after completing the Flourishing Check), or the waitlist-control group (which will continue their usual activities and only gain access to the Pathway Tool after post-test assessments are completed or the final data collection time point has closed).

Quantitative and qualitative data will be collected at five timepoints: [T1] pre-intervention (baseline); [T2] acceptability and feasibility questionnaires (intervention group only), 1 week after T1; [T3] test–retest reliability (n = 200), 2–4 weeks after T1; [T4] qualitative feedback on the Flourishing Check and Pathway Tool (*n* ≈ 30, intervention group only), 3 months after T1; and [T5] post-intervention outcome measures, 6 months after T1. See Table 1 for a summary of the research procedure.

### 2.2. Participants

Participant eligibility criteria are as follows: (1) must be a parent or primary caregiver of a child aged 0–5 years, (2) reside within the Perth metropolitan area, (3) be over 18 years of age, and (4) have reliable internet access. Parents who meet these criteria will be eligible to register one child per family in the study.

This study has been approved by The University of Western Australia (UWA) Human Research Ethics Committee (HREC) (Project No. 2025/ET000152) and is registered with the Australian New Zealand Clinical Trials Registry (ACTRN12626000187347). This study is a sub-project of ORIGINS.

Participants will be compensated for their time with e-gift cards (40 AUD at T1 and 20 AUD at all other timepoints). Focus group participants will be compensated 50 AUD per hour. Participants will receive an email prompt to complete outcome measures at six months’ post-intervention [T5] and will receive reminders after one and three weeks of non-completion. Participants who have not completed the questionnaires at T2 and T5, two weeks after the second reminder, will be marked as incomplete.

Participant communications will emphasise that the intervention is under development and results are not diagnostic. Phone numbers for support services will be provided to alleviate any concerns parents may have after completing the Flourishing Check. We do not anticipate any other unintended harms.

#### 2.2.1. Recruitment

A link to an Expression of Interest (EOI) will be sent to ORIGINS participants and community members by The Flourishing Child research team and partner organisations via email. Digital and printed promotional materials will be circulated through online platforms, social media, advertising, and within the local community. The EOI will direct participants to online study information (e.g., eligibility criteria, time commitment, reimbursement). After reviewing this information, participants who wish to take part will complete a screening form and, if eligible, provide electronic informed consent. We aim to recruit participants primarily within the Joondalup and Wanneroo regions over the three-month recruitment period, but we will also accept participants in the wider Perth metropolitan area.

Participants will access the intervention via personal devices. After eligibility screening, all participants will be randomly assigned to either a waitlist-control or intervention group using REDCap’s randomisation module, ensuring that assignment is concealed from researchers until after enrolment [41,42]. Randomisation will be conducted using a 2:1 allocation ratio (intervention/control), stratified by child age, gender, and locality. Participants will be notified of their group assignment and respective study involvement via email. Blinding cannot be implemented in this study, as participants would be aware of their group allocation due to the nature of the intervention; moreover, blinding is not required in this non-clinical trial context. All participants will complete a questionnaire battery at baseline. The intervention group will gain access to the Pathway Tool after completing the Flourishing Check. On completion of the full intervention (i.e., Flourishing Check and Pathway Tool), we will ask intervention group participants to register their interest in participating in focus groups. The waitlist-control group will continue their usual activities. Once data collection has been completed, they will be given access to the Pathway Tool. For ethical reasons, waitlist control groups are preferred over no-treatment controls [43]. Recent trials testing digital parenting interventions have used waitlist controls [44,45,46]. For these reasons, a waitlist control will be used in this study.

#### 2.2.2. Attrition Management

Automated survey invitations will be configured in REDCap (Version 16.1.4) [42,43] to issue reminder notifications at 7 and 14 days for participants with incomplete follow-up surveys at T2 and T3. The ‘Save and Return later’ feature in REDCap will be enabled to enhance participant retention and reduce response burden by allowing surveys to be completed in multiple sittings. Incentives will only be offered upon completion of all questionnaires.

### 2.3. Measures

#### 2.3.1. Pathway Tool Acceptability and Feasibility Questionnaire

An author-developed survey will be used to evaluate the acceptability and feasibility of the Pathway Tool. The survey, adapted from the PEMAT (Patient Education Materials Assessment Tool) framework [47,48], will measure the ease of understanding, visual appeal, acceptability of language, readability, functionality, and ease of navigation of the Pathway Tool (intervention group only). See Section S4 of the Supplementary Materials for further details on this measure.

#### 2.3.2. Pathway Tool Engagement Metrics

Engagement metrics will be used to provide insights into how participants are using the Pathway Tool and guide improvements to make it more user-friendly. These metrics will be captured during participant use of the Pathway Tool. See Table 2 for engagement metrics and operational definitions.

#### 2.3.3. Parent Outcome Measures

Questionnaires developed by the authors will assess pre-to-post-intervention changes in (i) perceived self-efficacy in recognising and supporting flourishing in early childhood, (ii) perceived knowledge of how and when to seek support, and (iii) engagement with and participation in supports relevant to flourishing in the early years. These questionnaires will include items such as “I can tell when my child is doing well” and “I know about good programmes and services in my area that can help my child flourish”. The questionnaire items were developed by the research team and reviewed by experts in child wellbeing, child development, and questionnaire design. For further details, see Section S3 of the Supplementary Materials.

Parent wellbeing will be assessed pre- and post-intervention using the Mental Health Continuum–Short Form (MHC-SF) [49]. This scale measures three dimensions of mental health: emotional, psychological, and social wellbeing, and shows acceptable internal consistency (α = 0.89), test–retest reliability (0.65–0.70, *p* < 0.001), and low–moderate convergent validity in expected directions [50]. The Paediatric Quality of Life Inventory (version 4.0) (PedsQL 4.0) [51], which measures the health-related quality of life of children and adolescents, will be used for a health economics analysis. This analysis will quantify any improvements attributable to the Flourishing Intervention in terms of cost-effectiveness. This will enable the assessment of whether the intervention delivers meaningful improvements in child wellbeing relative to its costs, such as the incremental cost per improvement in quality-adjusted life years.

We do not anticipate significant improvements to parental wellbeing or paediatric quality of life within the timeframe of this study. Completion of these measures will be used to inform a future effectiveness trial.

#### 2.3.4. Focus Groups

A subset of participants (approximately n = 30) will participate across two focus groups to report on how they used the Pathway Tool and their experiences and engagement with the supports. Focus group questions have been developed in collaboration with members of The Flourishing Child Consumer and Community Advisory Group. To achieve saturation, we will follow procedures recommended by Krueger and Casey [49]. They advise recruiting smaller groups of five to eight participants in focus groups when dealing with topics that may be sensitive and/or are experienced by the participants. Usually, saturation is achieved after completing three to four groups. We will conduct two focus groups before reviewing responses to assess whether the questions are eliciting responses that are relevant to our aims. We will then amend questions if required and conduct two more focus groups. We will recruit five to eight participants into each group. We will stratify those who register by demographics and other factors (e.g., parents of children with additional challenges) and invite them to participate in either online or in-person focus groups.

All focus group participants will receive an e-gift voucher of $50 for their contribution. Focus groups will be recorded and audio-transcribed. Qualitative data collected during focus groups will be analysed to identify key themes regarding programme engagement, acceptability, and confidence in accessing support and resources.

### 2.4. Data Collection and Management

Study data will be collected and managed using REDCap electronic data capture tools hosted at The Kids Research Institute Australia [43]. REDCap (Research Electronic Data Capture) is a secure, web-based software platform designed to support data capture for research studies, providing (1) an intuitive interface for validated data capture, (2) audit trails for tracking data manipulation and export procedures, (3) automated export procedures for seamless data downloads to common statistical packages, and (4) procedures for data integration and interoperability with external sources. Participants will be issued a unique identifier, and all data will be deidentified. When participants complete T1 data collection in REDCap, their Flourishing Check results, child’s date of birth, and sex data will be synced to the data platform hosted within an Amazon Web Services account owned by ORIGINS. This will enable Flourishing Check results to inform which resources are recommended by the Pathway Tool. The Pathway Tool will link to the data platform only (there will be no direct connection to REDCap). A data management committee (DMC) will not be established, and no interim analysis or formal stopping rules are planned for this trial due to its low risk and short duration. Any adverse events associated with this research will be reported to UWA HREC according to standard procedures.

### 2.5. Data Screening

Data screening will be automated using formulae within REDCap’s data quality functionality. Participant records will be flagged where responses are inconsistent or contradictory, attention checks are failed, or key variables are missing responses. As an additional measure, records will be manually screened by members of the research team to validate participants. Data will be monitored by research team members throughout the recruitment and follow-up periods to ensure that (1) recruitment targets are achieved, (2) participants are reimbursed appropriately and efficiently, and (3) only valid data is included in the analysis.

### 2.6. Data Analysis

#### 2.6.1. Primary Outcomes

Acceptability and Feasibility:

The acceptability and feasibility of the Flourishing Check and Pathway Tool will be assessed using a convergent mixed-methods design [52] involving data from questionnaires and focus groups. Additional themes emerging from focus groups, as well as website engagement metrics, will inform future modifications to these tools. Convergent or concurrent mixed methods (also called “parallel”) projects gather quantitative and qualitative data separately, or parallel to each other, and analyse them together. All quantitative and qualitative data will be triangulated and compared for consistency, validity, and credibility. The results will be synthesised to identify themes and recommendations for improving the intervention. Table 3 provides a summary of the data triangulation process and criteria to meet different levels of feasibility and acceptability. This has been created based on the ADePT framework, where 3 categories (green, amber, or red) are used to assess whether a study should progress from a pilot to an efficacy trial [53].

Questionnaires:

The author-developed questionnaires will assess the ease of use, understanding, visual appeal, acceptability of language, readability, functionality, and ease of navigation of the Flourishing Check and Pathway Tool, with higher scores operationalised as greater acceptance. These data will be analysed using descriptive statistics. Additionally, responses to open-ended questions will be subjected to thematic analysis according to best practice, described below [54].

Focus Groups:

Focus groups will be used to assess the feasibility and acceptability of the Pathway Tool. Using the approach of Braun and Clarke [54] for thematic analysis, transcripts will be iteratively coded, and codes will be collated into higher-level themes. Transcripts will be reviewed to identify all instances of thematic codes, with codes expanded or collapsed as required. Qualitative data will be analysed using qualitative content analysis [55], assigning a code to each concept using NVivo 14. Similar concepts will be identified and categorised into categories. Data will be analysed using a phenomenological approach (i.e., as a description of experiences as consciously experienced by participants), and narrative themes will be deducted until saturation. The coding process will be guided by the following deductive constructs: intervention acceptability, engagement, and application; mental health and wellbeing experiences; and suggestions for programme revisions.

Engagement metrics:

This data will be used for exploratory purposes to understand how users interact with suggested resources. Exploratory engagement (e.g., clicking resources within the tool or following external links) will indicate whether recommendations are perceived as relevant and worth investigating, supporting acceptability. Recurring engagement, such as saving resources, will indicate sustained interest and practical feasibility of repeated use over time. Collaborative engagement (i.e., copying links to share with others) will further signal acceptability by indicating that users value the content enough to recommend it within their community. These metrics will inform future modifications to the Pathway Tool if the feasibility or acceptability receives an ‘amber’ outcome (see Table 3).

#### 2.6.2. Secondary Outcomes

Parent and Child Outcomes:

All participants (intervention and control) will be asked to repeat the battery of measures completed at T1 to assess if there has been any change over time (within groups) and/or as a result of the Pathway Tool (between groups). Specifically, we will explore the direction and magnitude of any changes to (i) perceived self-efficacy in recognising and supporting flourishing in early childhood, (ii) perceived knowledge of how and when to seek support, (iii) engagement with and participation in supports relevant to flourishing in the early years, (iv) parent wellbeing, and (v) paediatric quality of life.

Data will be described for each available time point using frequencies and proportions for categorical data, medians and interquartile ranges for skewed data, and means and standard deviations for data that is normally distributed.

Outcomes will be analysed using mixed-effects regression models to account for repeated measures within participants. For continuous and Likert-scale outcomes, linear mixed-effects models will be used. In the presence of substantial skewness or deviation from model assumptions, alternative approaches such as mixed-effects quantile regression will be considered. For binary outcomes, generalised linear mixed-effects models with a binomial distribution and logit link function will be used. All models will include fixed effects for treatment group, time (baseline and follow-up), and their interaction, with a random intercept for participants. Models will be adjusted for the covariates used in the stratified randomisation, which include child age, gender, and locality, along with potential confounders, including socioeconomic status and mother’s age. Maternal age and socioeconomic status were included as covariates due to their established associations with both intervention engagement and outcomes related to parental wellbeing and child quality of life. Adjusting for these variables aimed to improve the precision of estimates and account for potential residual imbalance between groups.

When exploring these between-group differences, baseline values of the outcome will be included as covariates to allow adjustment for baseline score. Subgroup analysis by child age group will be conducted (0–2 and 3–5 years). We will report the precision of the model estimated by providing 95% confidence intervals. Missing outcome data will be handled under the assumption of missing at random using likelihood-based estimation inherent in mixed-effects models. Sensitivity analyses will be conducted to assess the robustness of findings, including analyses based on multiple imputation and complete-case analysis.

It has been previously acknowledged that the secondary outcome measures are considered exploratory data, with only small changes anticipated. Although statistical tests will be conducted, the focus will be on the direction and magnitude of change.

Sample Size:

We will aim to recruit N = 600 participants, with n = 400 randomly assigned to the intervention group and n = 200 to the control group. The primary acceptability endpoint is defined as the proportion of participants who rate all key acceptability items as ≥4. This outcome is therefore treated as a binomial proportion. The sample size was chosen to ensure adequate precision around the estimated proportion. Assuming an expected proportion of 60% (based on the predefined acceptability threshold), a sample size of 369 participants, which we rounded up to 400, yields a two-sided 95% confidence interval with a margin of error of approximately ±5%. Unequal allocation (2:1) was intentionally chosen to maximise the amount of information obtained on feasibility and acceptability outcomes in the intervention group, which is the primary focus of the study. The control group remains sufficiently sized to provide a comparator and to inform the design of a future definitive trial. While comparisons between groups will be conducted, these analyses will be considered exploratory, with emphasis placed on the direction and magnitude of effects rather than formal hypothesis testing. After allowing for a loss to follow up of approximately one third in each group, a total sample size of N = 400 would be available for follow-up analysis (intervention group n = 267 and control n = 133). Exploration of any differences between groups in perceived self-efficacy in recognising and supporting flourishing in early childhood and knowledge of programmes and resources, would allow for a small effect size (*d* = 0.30) to be detected using a Mann–Whitney U test, with a power of 80% and alpha of 0.05.

#### 2.6.3. Triangulation of Data

**Figure 3:**
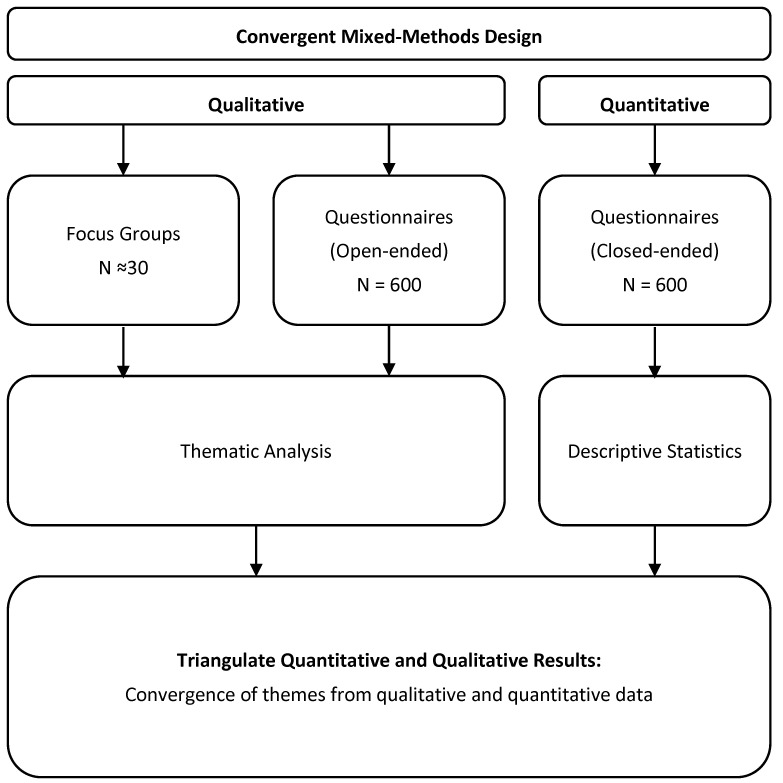
Visual summary of data triangulation.

The totality of the quantitative and qualitative data will be triangulated and interpreted together. We anticipate that the quantitative data will mainly provide insights into how people engaged with the intervention, whereas the qualitative data will provide insights into why they felt/acted this way. We will systematically compare and integrate the findings to assess where they converge or diverge. This will enable a more robust and holistic understanding of the Flourishing Intervention tools and inform further refinements to proceed to the next phase of an effectiveness trial. If the data sets are divergent, and depending on the extent of the divergence, the research team will undertake several strategies to address the divergence, including (a) reconciliation—a re-review of the raw data; (b) bracketing—acknowledging the conflict and/or separating the analyses and contextualising; and (c) complementary explanation—using the divergence to explain the complexity of a phenomenon. Furthermore, engagement metrics will also provide a more objective snapshot of Pathway Tool use if divergent findings are reported between the qualitative and quantitative datasets. For a visual summary of data triangulation, see Figure 3.

### 2.7. Dissemination of Results

The results of this study will be disseminated through peer-reviewed publications, presentations at national and international conferences, and plain language summaries sent to participants who opt to receive information about study outcomes. Findings will be circulated transparently regardless of the direction and significance of results.

## 3. Expected Results

We hypothesise that the Flourishing Intervention (Flourishing Check and Pathway Tool) will be acceptable and feasible to parents of children aged 0–5 years, as indicated by questionnaire responses and qualitative feedback (primary aim). Given the brief duration of the intervention and follow-up period, and the relative stability of many parenting and wellbeing characteristics over short timeframes, we anticipate detecting only small changes in most of the secondary outcome variables. We do not anticipate that we will detect significant improvements to parental wellbeing or paediatric quality of life within the timeframe of this study. However, this will be measured to assess the feasibility and sensitivity of these measures, and to inform effect size estimates and outcome selection for a future trial evaluating the intervention’s effectiveness.

## 4. Discussion

The protocol details a procedure to test the acceptability and feasibility of the Flourishing Intervention, a new digital intervention designed to promote early childhood and family flourishing. The primary aim is to assess whether the Flourishing Intervention is feasible and acceptable to parents and the secondary aims are to explore the potential efficacy of the intervention in improving (i) perceived self-efficacy in recognising and supporting flourishing in early childhood, (ii) perceived knowledge of how and when to seek support, (iii) engagement with and participation in supports relevant to flourishing in the early years, (iv) parental wellbeing, and (v) paediatric quality of life. It is hypothesised that the intervention in this pilot trial will be evaluated as feasible and acceptable based on the integration of qualitative and quantitative data.

This will be the first study to test the acceptability and feasibility of an early childhood flourishing assessment and digital intervention providing recommendations to local and national evidence-based supports. Parents are utilising online sources of information more frequently, but also report feeling overwhelmed by the volume of information available to them [56,57]. Online information comes from a wide variety of sources, increasing its potential to be conflicting and unreliable [58].

Parents need tools that reduce the burden of identifying the most appropriate support for their children [10]. The Flourishing Intervention has the potential to benefit parents and children by (1) reducing the time spent finding support, (2) improving parental awareness of their children’s strengths and support needs, and (3) promoting engagement with evidence-based programmes, services, and resources which promote flourishing.

Digital parenting interventions targeting early childhood aim to improve child and parent outcomes; however, many prioritise reducing child problem behaviours rather than enhancing strengths [16,19,24]. The Flourishing Intervention was developed through rigorous codesign with a range of primary and secondary stakeholders [10] and aims to promote flourishing by addressing problems and enhancing strengths.

Improving childhood health and wellbeing globally is a recognised imperative [1,2,3,4,5,6,7]. By increasing parents’ awareness of their children’s flourishing and knowledge of how to access support at a local level, this broader project (the feasibility and acceptability trial followed by an efficacy trial and scale-up) may inform methods for promoting early childhood flourishing at a larger scale.

### 4.1. Strengths and Limitations

#### 4.1.1. Strengths

There are multiple strengths to this protocol. A key strength of this methodology is its extensive and ongoing co-design with the community [10] and expert advisory groups. The resulting intervention is more likely to be engaging, acceptable, feasible, and meaningful to users [59].

A mixed-methods triangulation design will be utilised in the analyses. This approach was chosen as quantitative and qualitative methods can complement one another and together provide a more robust analysis [60]. By elevating parents’ voices through focus groups, this study will continue to engage in meaningful co-design with intended end-users and empower consumers [61]. The use of website metrics to track user engagement will allow for an in-depth understanding of how users engage with the Pathway Tool. This will allow the research team to improve the tool in future iterations.

#### 4.1.2. Limitations

There are limitations within this study that should be acknowledged. Participants will be self-selected from the Perth metropolitan area, with a focus on the Joondalup and Wanneroo regions. This may limit our ability to generalise conclusions about the acceptability and feasibility of the Flourishing Intervention beyond this area and to parents who are less likely to engage with digital tools. Additionally, all measures, barring engagement metrics, rely on self-report or parent proxy reports. Therefore, results may be distorted by biases or responder characteristics. Where possible, parenting outcome items included objective indicators (e.g., through questions about engagement with specific programmes) (see Section S3 of the Supplementary Materials for further details). While measurement of wellbeing in children under five years necessarily relies on parent reports [38], future research should incorporate objective measures and, where feasible, include the voice of the child. Additionally, participants will not be blinded to the condition they are allocated to, and those in the control condition may find a way to access the Pathway Tool (though this will be partly prevented by keeping the URL hidden from searches). If this occurs, this could potentially interfere with the engagement metrics recorded.

Lastly, the Consumer and Community Advisory Committee strongly agreed that enforcing user registration may create a barrier to engagement with online content. Therefore, participants will not have to create a user account to engage with the Pathway Tool. This limits our ability to control who accesses the tool, as anyone who receives the URL can access it. Therefore, engagement metrics may be inflated by users who are not verified, participants. Additionally, control participants may gain access to the tool during the study. This will be partly mitigated by the URL being hidden from searches. Given the low-risk nature of the intervention and the study context, more intensive monitoring approaches (e.g., individual-level tracking, mandatory logins, or detailed analytics linkage) were considered disproportionate to the aims of the study and not feasible within available resources. Importantly, such approaches would also introduce additional privacy considerations and participant burden, which may further impact participation and engagement. Future research could implement stricter controls to more accurately assess the efficacy of the Pathway Tool.

### 4.2. Outcomes of Research and Next Steps

If the Flourishing Child Intervention is assessed as acceptable and feasible by parents, it will provide accessible means for parents to gauge their child’s mental and physical health and engage targeted support when needed. The next phase of this project will test the effectiveness of the intervention in improving parent outcomes and early childhood flourishing. Insights gained through the feasibility trial will inform amendments to recruitment strategies, methods for maximising adherence and completion rates, data screening processes, and delivery of the intervention. Results from focus groups, questionnaires, and website metrics will inform refinements of the intervention itself to maximise utility and reduce participant burden.

Subsequent research will focus on implementing the intervention at scale in different geographical settings and with different population groups, initially in Western Australia. This will involve co-design with local consumers and contextual and cultural adaptation, ensuring acceptability and feasibility among different populations, including Culturally and Linguistically Diverse (CALD) families, Aboriginal and Torres Strait Islanders, families with neurodiverse children, and other groups [13]. In scaling up this digital intervention, the research team will contextualise the geographical region and local population, including digital access, literacy, and connectivity.

Future research will include a cost-effectiveness analysis comparing those who utilise the Pathway Tool and those who do not, as well as health service utilisation and associated costs. Project impacts will incorporate key recommendations to be articulated in a policy briefing paper. Findings will be disseminated widely through publications and stakeholder presentations.

Early childhood parenting interventions have tended to focus on reducing child problem behaviours [16,19,24]. The Flourishing Intervention is unique in its focus on enhancing the positive aspects of a child’s life. The Flourishing Intervention has the potential to increase parents’ awareness of their children’s strengths and support needs, and confidence in supporting them to flourish. According to the Logic Model provided in Figure 1, we anticipate the potential for long-term positive impacts as a result of parents engaging with the Flourishing Intervention (such as reduced incidence of mental health issues in children).

## Figures and Tables

**Table 1:** Schedule of enrolment, interventions, and assessments.

	STUDY PERIOD							
	Enrolment	Allocation	Post-Allocation					Close-Out
TIMEPOINT	−*t*_1_	0	*t*_1_	*t*_2_	*t*_3_	*t*_4_	*t*_5_	*t_x_*
**ENROLMENT**:								
Eligibility screen	X							
Informed consent	X							
Allocation		X						
**INTERVENTIONS**:								
Intervention Group								
Flourishing Check			X		X ^†^			
Pathway Tool								
Waitlist Control Group								
Flourishing Check			X		X ^†^			
Pathway Tool			X **—————————————→** X					X
**ASSESSMENTS**:								
Demographics			X					
Flourishing Check Acceptability and Feasibility Questionnaire ^†^			X					
Pathway Tool Acceptability and Feasibility Questionnaire ^×^*				X				
Focus groups *						X		
Perceived self-efficacy in recognising and supporting flourishing in early childhood ^×^			X				X	
Perceived knowledge of how and when to seek support ^×^			X				X	
Engagement with and participation in supports relevant to flourishing in the early years ^×^			X				X	
Mental Health Continuum–Short Form (MHC-SF)			X				X	
Paediatric Quality of Life (PedsQL)			X				X	

* Only the intervention group will complete these assessments. ^×^ Can be found in Section S3 and S4 of the Supplementary Materials. ^†^ Can be found in Section S5 of the Supplementary Materials. The analysis plan and results pertaining to these measures (i.e., test–retest reliability measured at T3 and Flourishing Check Acceptability and Feasibility Questionnaire measured at T1) will be detailed in a separate paper explaining the development of the Flourishing Check.

**Table 2:** Description of Pathway tool engagement metrics.

Engagement Type	Engagement Category	Metric	Description
Passive Information Access	Exploratory Engagement	Level 1–A resource clicked within the Pathway Tool Level 2–Participant follows link to external resource	An indicator over two levels that the participant finds the resource engaging and relevant
Active Information Access	Recurring Engagement	Total number of resources saved	An indicator that participant is interested in engaging with the resource more than once
Community Participation	Collaborative Engagement	A resource link copied to share	An indicator that the participant wants others in the community to engage with the resource

**Table 3:** Data triangulation process and criteria used to assess feasibility and acceptability.

	Red (Do Not Proceed to Full-Scale RCT)	Amber (Consider Proceeding to Full-Scale Trial Only After Intervention and Protocols Have Been Refined)	Green (Proceed to Full-Scale RCT Without Refinement)
Flourishing Check	<40% of participants rated all key acceptability items as ≥4 AND Focus groups revealed repeated themes of low to moderate acceptability	40–60% of participants rated all key acceptability items as ≥4 AND Focus groups revealed repeated themes of moderate to high acceptability	>60% of participants rated all key acceptability items as ≥4 AND Focus groups revealed repeated themes of high acceptability
Pathway Tool	<40% of participants rated all key acceptability items as ≥4 AND Focus groups revealed repeated themes of low to moderate acceptability	40–60% of participants rated all key acceptability items as ≥4 AND Focus groups revealed repeated themes of moderate to high acceptability	>60% of participants rated all key acceptability items as ≥4 AND Focus groups revealed repeated themes of high acceptability
Flourishing Check	<40% of participants rated all key feasibility items as ≥4 AND Focus groups revealed repeated themes of low to moderate feasibility	40–60% of participants rated all key feasibility items as ≥4 AND Focus groups revealed repeated themes of moderate to high feasibility	>60% of participants rated all key feasibility items as ≥4 AND Focus groups revealed repeated themes of high feasibility
Pathway Tool	<40% of participants rated all key feasibility items as ≥4 AND Focus groups revealed repeated themes of low to moderate feasibility	40–60% of participants rated all key feasibility items as ≥4 AND Focus groups revealed repeated themes of moderate to high feasibility	>60% of participants rated all key feasibility items as ≥4 AND Focus groups revealed repeated themes of high feasibility

Note: For each feasibility and acceptability outcome, analyses will be stratified by age group (0–2 years and 3–5 years). See Section S6 of the Supplementary Materials for further details of key acceptability and feasibility items.

## Data Availability

No data was used for this article. Protocol version 6.0 (2 April 2026) updated following peer review.

## Supplementary Materials

The following supporting information can be downloaded at https://www.mdpi.com/article/10.3390/children13050581/s1: Section S1. Pathway Tool Development Process; Section S2. SPIRIT 2025 checklist of items to address in a randomized trial protocol; Section S3. Questionnaires Assessing Parenting Outcomes; Section S4. Pathway Tool Acceptability and Feasibility Questionnaire; Section S5. Flourishing Check Acceptability and Feasibility Questionnaire; Section S6. Key and Exploratory Acceptability and Feasibility Items for Evaluation of the Pathway Tool and Flourishing Check.

## Abbreviations

The following abbreviations are used in this manuscript:

WHO	World Health Organization
ECI	Early Childhood Intervention
UWA	University of Western Australia
HREC	Human Research Ethics Committee
EOI	Expression of Interest
IG	Intervention Group
CG	Control Group
PEMAT	Patient Education Materials Assessment Tool
CALD	Culturally and Linguistically Diverse
