# Supplementary material for: The Flourishing Child: Study Protocol for an Acceptability and Feasibility Trial of a Digital Early Childhood Flourishing Intervention
Source: Children (Basel). 2026 Apr 22;13(5):581. doi: 10.3390/children13050581 (PMC13204361; doi:10.3390/children13050581)
Supplement: Supplementary file 1 [file children-13-00581-s001.zip › children-4220368-supplementary.pdf]

Appendix A: Pathway Tool Development Process

	**Development Stage**	**Method**
**Content Development**	Define Inclusion/ Exclusion Criteria	▪ Inclusion Criteria:<br>1. Updated in the previous 12 months<br>2. Linked to early childhood flourishing as defined by our framework[10]<br>3. Resources are verifiably Australian<br>4. Programs are accessible to residents of the Joondalup and Wanneroo regions of Perth, WA
	Initial Search	▪ Google Advanced searches were conducted to identify programs, services and resources that support early childhood flourishing as defined by our framework.<br>▪ Each search string contained terms relevant to three key concepts: target group, content, and region, as well as terms relevant to flourishing.<br>▪ 29 searches produced 4,535 results. 2,769 duplicates were removed, leaving 1,766 results for review.
	Initial Eligibility Screen	▪ Results were independently screened by two reviewers who demonstrated an acceptable level of inter-rater agreement (*k*=0.86) from a sample of 200 results.<br>▪ 25 disagreements were resolved through discussion between reviewers and input from a third reviewer for unresolved disagreements.<br>▪ 335 (19%) results were identified as eligible for inclusion in the Pathway Tool.
	Data Extraction	▪ Data on the resource or program (including name, URL, delivery mode, contact details and availability) were extracted for each included result into an excel spreadsheet.
	Develop Quality Assessment Framework	▪ Quality Assessment (QA) Criteria were developed based on:<br>a. Existing methods[49]<br>b. Existing frameworks (e.g., for early childhood services)<br>c. Relevance to the early childhood flourishing framework<br>▪ Two rubrics were developed with criteria assessing:<br>1. Programs and services and<br>2. Resources that support or facilitate early childhood flourishing.
	Apply Quality Assessment Framework	▪ A single reviewer (JK) assessed quality of eligible search results against all applicable criteria.<br>▪ Any programs and/or services falling below a cutoff score for the evidence-base criterion were excluded.

		▪ Any resources falling below a cutoff score for the credibility criterion were excluded.<br>▪ 154 items passed QA and were included in the Pathway Tool.
	Additional Items	▪ Where a single provider offered multiple services, programs or resources that were eligible and passed QA, these items were extracted and included in the Pathway Tool separately<br>▪ Targeted manual searches (hand-searches) were conducted to identify items that were not adequately captured by the systematic search.<br>▪ Items eligible for inclusion were also identified during QA of systematic search results (e.g., linked resources within search result items).<br>▪ An additional 105 items were identified through hand-searches and expert recommendation. From these, 73 passed quality assessment.<br>▪ Google maps searches were conducted to ensure coverage of local programs and services falling under relevant categories (e.g., swim schools, sports programs, antenatal education).<br>▪ 58 items were identified as eligible and 35 passed QA.
**Website Development**	Website Development	▪ Researchers and web-developers collaborated to ensure the Pathway Tool design and structure was user friendly and consistent with the researchers aims.<br>▪ The structure of the tool was developed within Payload, an open-source headless CMS and application framework.
	Content Upload	▪ A research assistant manually imported data previously extracted from 250 included programs, services and resources into the Pathway Tool using Payload's admin area. These data included:<br>a. Brief descriptions of the program, service or resource<br>b. Relevant keywords, manually extracted from the source code of the resource, to improve search functionality<br>c. Data extracted as above (e.g. availability, cost, contact information)
	User Acceptance Testing (UAT)	▪ UAT was run by the web-developers, based on human centred design to identify issues in the tool experienced by intended end-users.<br>▪ Interested members (*n*=8) of the Consumer and Community Advisory Committee accessed and explored the tool and provided feedback via a questionnaire implemented in REDCap.<br>▪ Consumers completed questionnaires assessing functionality & design of tool, workflows, visualisation of results, useability of viewing resources, simplicity of navigation, programs listed and their overall experience.

		▪ Feedback was received and implemented by web-developers.
	Review by Research Team	▪ Team meetings were held for a final review of the content and design of the tool to identify changes to be implemented prior to commencing acceptability and feasibility testing.

Appendix B: SPIRIT 2025 checklist of items to address in a randomized trial protocol

Section / Topic	No	SPIRIT 2025 checklist item description	Reported on page no.
**Administrative information**			
Title and structured summary	1a	Title stating the trial design, population, and interventions, with identification as a protocol	1
	1b	Structured summary of trial design and methods, including items from the World Health Organization Trial Registration Data Set	2,6 - 8
Protocol version	2	Version date and identifier	17
Roles and responsibilities	3a	Names, affiliations, and roles of protocol contributors	1
	3b	Name and contact information for the trial sponsor	1,6
	3c	Role of trial sponsor and funders in design, conduct, analysis, and reporting of trial; including any authority over these activities	17
	3d	Composition, roles, and responsibilities of the coordinating site, steering committee, endpoint adjudication committee, data management team, and other individuals or groups overseeing the trial, if applicable	6
**Open science**			
Trial registration	4	Name of trial registry, identifying number (with URL), and date of registration. If not yet registered, name of intended registry	7
Protocol and statistical analysis plan	5	Where the trial protocol and statistical analysis plan can be accessed	6-9
Data sharing	6	Where and how the individual de-identified participant data (including data dictionary), statistical code, and any other materials will be accessible	17
Funding and conflicts of interest	7a	Sources of funding and other support (e.g., supply of drugs)	17
	7b	Financial and other conflicts of interest for principal investigators and steering committee members	17

Dissemination policy	8	Plans to communicate trial results to participants, healthcare professionals, the public, and other relevant groups (e.g., reporting in trial registry, plain language summary, publication)	14
**Introduction**			
Background and rationale	9a	Scientific background and rationale, including summary of relevant studies (published and unpublished) examining benefits and harms for each intervention	2-6
	9b	Explanation for choice of comparator	8
Objectives	10	Specific objectives related to benefits and harms	6
**Methods: Patient and public involvement, trial design**			
Patient and public involvement	11	Details of, or plans for, patient or public involvement in the design, conduct, and reporting of the trial	3
Trial design	12	Description of trial design including type of trial (e.g., parallel group, crossover), allocation ratio, and framework (e.g., superiority, equivalence, non-inferiority, exploratory)	6
**Methods: Participants, interventions, and outcomes**			
Trial setting	13	Settings (e.g., community, hospital) and locations (e.g., countries, sites) where the trial will be conducted	8
Eligibility criteria	14a	Eligibility criteria for participants	7
	14b	If applicable, eligibility criteria for sites and for individuals who will deliver the interventions (e.g., surgeons, physiotherapists)	NA
Intervention and comparator	15a	Intervention and comparator with sufficient details to allow replication including how, when, and by whom they will be administered. If relevant, where additional materials describing the intervention and comparator (e.g., intervention manual) can be accessed	5
	15b	Criteria for discontinuing or modifying allocated intervention/comparator for a trial participant (e.g., drug dose change in response to harms, participant request, or improving/worsening disease)	10
	15c	Strategies to improve adherence to intervention/comparator protocols, if applicable, and any procedures for monitoring adherence (e.g., drug tablet return, sessions attended)	8

	15d	Concomitant care that is permitted or prohibited during the trial	NA
Outcomes	16	Primary and secondary outcomes, including the specific measurement variable (e.g., systolic blood pressure), analysis metric (e.g., change from baseline, final value, time to event), method of aggregation (e.g., median, proportion), and time point for each outcome	10 -12
Harms	17	How harms are defined and will be assessed (e.g., systematically, non-systematically)	8
Participant timeline	18	Time schedule of enrollment, interventions (including any run-ins and washouts), assessments, and visits for participants. A schematic diagram is highly recommended (see Figure)	6
Sample size	19	How sample size was determined, including all assumptions supporting the sample size calculation	13
Recruitment	20	Strategies for achieving adequate participant enrollment to reach target sample size	8
**Methods: Assignment of interventions**			
Randomization:			
Sequence generation	21a	Who will generate the random allocation sequence and the method used	8
	21b	Type of randomization (simple or restricted) and details of any factors for stratification. To reduce predictability of a random sequence, other details of any planned restriction (e.g., blocking) should be provided in a separate document that is unavailable to those who enroll participants or assign interventions	8
Allocation concealment mechanism	22	Mechanism used to implement the random allocation sequence (e.g., central computer/telephone; sequentially numbered, opaque, sealed containers), describing any steps to conceal the sequence until interventions are assigned	8
Implementation	23	Whether the personnel who will enroll and those who will assign participants to the interventions will have access to the random allocation sequence	8
Blinding	24a	Who will be blinded after assignment to interventions (e.g., participants, care providers, outcome assessors, data analysts)	8

	24b	If blinded, how blinding will be achieved and description of the similarity of interventions	8
	24c	If blinded, circumstances under which unblinding is permissible, and procedure for revealing a participant's allocated intervention during the trial	NA
**Methods: Data collection, management, and analysis**			
Data collection methods	25a	Plans for assessment and collection of trial data, including any related processes to promote data quality (e.g., duplicate measurements, training of assessors) and a description of trial instruments (e.g., questionnaires, laboratory tests) along with their reliability and validity, if known. Reference to where data collection forms can be accessed, if not in the protocol	10
	25b	Plans to promote participant retention and complete follow-up, including list of any outcome data to be collected for participants who discontinue or deviate from intervention protocols	7-8
Data management	26	Plans for data entry, coding, security, and storage, including any related processes to promote data quality (e.g., double data entry; range checks for data values). Reference to where details of data management procedures can be accessed, if not in the protocol	10
Statistical methods	27a	Statistical methods used to compare groups for primary and secondary outcomes, including harms	10-12
	27b	Definition of who will be included in each analysis (e.g., all randomized participants), and in which group	10-12
	27c	How missing data will be handled in the analysis	12
	27d	Methods for any additional analyses (e.g., subgroup and sensitivity analyses)	12-13
**Methods: Monitoring**			
Data monitoring committee	28a	Composition of data monitoring committee (DMC); summary of its role and reporting structure; statement of whether it is independent from the sponsor and funder; conflicts of interest and reference to where further details about its charter can be found, if not in the protocol. Alternatively, an explanation of why a DMC is not needed	10

	28b	Explanation of any interim analyses and stopping guidelines, including who will have access to these interim results and make the final decision to terminate the trial	10
Trial monitoring	29	Frequency and procedures for monitoring trial conduct. If there is no monitoring, give explanation	10
**Ethics**			
Research ethics approval	30	Plans for seeking research ethics committee/institutional review board approval	7
Protocol amendments	31	Plans for communicating important protocol modifications to relevant parties	N/A
Consent or assent	32a	Who will obtain informed consent or assent from potential trial participants or authorized proxies, and how	8
	32b	Additional consent provisions for collection and use of participant data and biological specimens in ancillary studies, if applicable	NA
Confidentiality	33	How personal information about potential and enrolled participants will be collected, shared, and maintained in order to protect confidentiality before, during, and after the trial	10
Ancillary and post-trial care	34	Provisions, if any, for ancillary and post-trial care, and for compensation to those who suffer harm from trial participation	8

Appendix C: Questionnaires Assessing Parenting Outcomes

Perceived self-efficacy in recognising and supporting flourishing in early childhood

This questionnaire focuses on Flourishing in early childhood (0-5 years) and your confidence in recognizing and supporting flourishing in your child, specifically.

We define Early Childhood Flourishing as the extent to which a child experiences safety and security, love and connection, a positive lifestyle, wellbeing, fun and play, and curiosity. The below framework was developed through consultation with parents, carers, children and early childhood development experts and informed by a review of published literature on early childhood flourishing.

Early Childhood Flourishing Framework:

**Pre-conditions of Flourishing**	**Safety and Security**	The family has access to health care, material basics (food, water, toilet, clothing, internet) and lives in a safe and stable home. The child is physically, virtually and culturally safe.
	**Love and Connection**	The child has a loving emotional bond with their primary caregivers and those who care for them. The child has a positive connection with the community.
	**Positive Lifestyle**	The family environment supports healthy habits including being physically active, healthy eating, getting enough sleep, learning opportunities and a balanced approach to screen time.
**Attributes of Flourishing**	**Wellbeing**	The child is happy, bounces back from setbacks, and expresses independence and emotions, all in a developmentally appropriate way.
	**Fun and Play**	The child has fun playing indoors and outdoors, alone or with others.
	**Curiosity**	The child shows interest and curiosity in learning new things, exploring, engaging with their surroundings and making discoveries.

Please answer the following questions in relation to how you have felt over the **last month**.

Identifying childhood flourishing

[Response format: 1 Strongly disagree – 5 strongly agree]

a. I knew when my child experienced the following (in relation to the above diagram):
    i. Safety and security
    ii. Love and connection
    iii. A positive lifestyle
    iv. Wellbeing
    v. Fun and play
    vi. Curiosity
b. I could identify my child's strengths which enable them to flourish.
c. I knew what it looked like when my child was flourishing.

Identifying gaps in programs available

a. I wanted more services in my local area which supported the following flourishing domains
    i. Safety and security
    ii. Love and connection
    iii. A positive lifestyle
    iv. Wellbeing
    v. Fun and play
    vi. Curiosity

Recognising child support needs

a. I could recognise the signs that my child needed support.

Seeking help

a. I knew what support my child needs.
b. I knew <u>how</u> to seek support for my child.
c. I actively sought information which helped me support my child

Access to Supports

[Response format: Yes/No]

a. I was able to access support for my child (for example, local programs and services)
b. I was able access support for myself (for example, parenting programs, online resources)
c. I found it difficult to access support for my child or myself because of problems with: [Tick Box]
    i. Cost
    ii. Transport
    iii. Time
    iv. Access to childcare or other carers
    v. Other barriers (please specify)
    vi. I did not have difficulty

Participation in programs and supports

We would now like to ask about your participation in programs and supports that promote flourishing. When answering the following questions, please think about your participation since being involved in this study (i.e., the **previous 6 months**) and select all responses that apply.

1. Need for support
   a. In the past 6 months, have you felt that you or your child needed more access to programs or supports? (Select one option)
      i. No, we didn't need any more support
      ii. Yes, we needed support, and our needs were fully met
      iii. Yes, we needed support, and our needs were partly met
      iv. Yes, we needed support, but our needs were not met at all

[Skip logic: 1.a. iii or iv endorsed]

i. Could you tell us what type of support you felt you needed? (free text response)

2. In the past 6 months, have you participated in or engaged with any of the following programs or supports for your child or family for the first time? (Tick all that apply.)

[Response format: Yes/No radio buttons]

a. General Supports
   i. Playgroups (e.g., community-based, library programs)
   ii. Parenting programs or workshops (e.g., Triple P, Tuning in to Kids)
   iii. Child and family health services (e.g., child health nurse checks, nurse drop-ins)
   iv. Early literacy or school readiness activities (e.g., Storytime, Let's Read)
   v. Recreational programs (e.g., swimming programs, music or art classes)
   vi. Online parenting resources or webinars
   vii. Other (please specify): ___________________________

[Skip Logic: If **any** box answered yes under 2.a]

a. In the past 6 months, how often did you participate in *any of* these programs
   i. Weekly
   ii. Fortnightly
   iii. Monthly
   iv. Less often
   v. One-time only

b. Individual Supports
   viii. Early intervention services (e.g., speech or occupational therapy)
   ix. Family support services (e.g., home visiting, case management)
   x. Psychological services for parents or children (e.g., counselling, perinatal support)
   xi. Parent–child attachment programs (e.g., Circle of Security)
   xii. Other targeted supports (please specify): ___________________________

[Skip Logic: If **any** box answered yes under 2.b]

a. In the past 6 months, how often did you participate in *any of* these programs
   i. Weekly
   ii. Fortnightly

iii. Monthly
iv. Less often
v. One-time only

3. Did you participate in these supports before you were involved in this research study? (Select one option)
   a. No, we didn't participate in any of the above support or programs
   b. Yes, we participated in a few available supports/programs
   c. Yes, we participated in most available supports/programs
   d. Yes, we were already participating in all of these before the research study

4. <u>Intention to use supports</u>

[Response format: 1 Strongly disagree – 5 strongly agree]

   a. I plan to engage more with programs in my local area that could support my child to flourish.
   b. I have enrolled my child in programs that help them flourish.

   [Skip logic: If Strongly Disagree/Disagree to 4a]

   c. I do not intend to engage with programs because I believe:
      i. My child has enough support.
      ii. My family has enough support.
      iii. Other _____

Appendix D: Pathway Tool Acceptability and Feasibility Questionnaire

Thank you for completing the Flourishing Check and using the Pathway Tool. We would like to know about your experience with the Pathway Tool. Specifically, we would like to know if you found the information useful, and that you feel you could gain knowledge and complete actions after accessing it. Please read each statement carefully and choose the most appropriate response for all questions.

Since you were sent the link to the Pathway Tool, how often have you opened it to explore the resources?

a. Every day
b. A few times a week
c. Once a week

Response Scale*

Response Options				
Strongly disagree	Disagree	Neither agree nor disagree	Agree	Strongly agree
1	2	3	4	5

*Applies to all below subscales unless otherwise specified

1. I understand the purpose of the Pathway Tool.
2. The information in the Pathway Tool is relevant to me.
3. The Pathway Tool includes a good amount of information on:
    a. Online resources
    b. Face to face programs and/or services
4. The Pathway Tool contains a good balance of online resources and face to face programs and/or services.
5. The Pathway Tool contains a good amount of:
    a. Audio Resources
    b. Video Resources
    c. Written resources
6. The language in the Pathway Tool is easy for me to understand.
7. The different pages in the Pathway Tool website are easy to navigate.
8. The way resources are organised within the tool makes it easy to find what I need.
9. The information in the Pathway Tool is presented in an order that is easy to understand.
10. The search filters make it easy to find what I need.
11. The visual cues (e.g., icons, keywords) in the Pathway Tool draw my attention to important information.
12. The text on screen is easy to read.
13. The Pathway Tool helps me find resources and programs that are relevant to me or my child.
14. I believe the Pathway Tool can help me support my child/children.
15. I am likely to engage with services or programs I found through the Pathway Tool.
16. I am likely to engage with online resources I found through the Pathway Tool.
17. I am likely to use the Pathway Tool again in the future.
18. I can identify at least one action I can take based on the Pathway Tool.
19. Did you create a user account within the Pathway Tool? (Y/N) If you did not use any of these functions, please select the “NA” option.

[IF YES]

a. I was able to set up my user account easily.
b. I was able to save resources easily.
c. The feedback from the Flourishing Check within the Pathway Tool was helpful.
d. I was able to set up child profiles easily.
e. I found the ability to set up child profiles helpful.
f. I was able to add additional family members easily.
g. I found the ability to add additional family members helpful.

Open-ended items (response optional):
What type/s of resources or programs would you like to see **more of** in the Pathway Tool?
What type/s of resources or programs would you like to see **less of** in the Pathway Tool?
Do you have any other feedback on the Pathway Tool?

Appendix E: Flourishing Check Acceptability and Feasibility Questionnaire

We would now like to ask you some final questions about your experience completing the Flourishing Check. Your feedback is highly valuable and will help us enhance the Flourishing Check for future use by parents and carers. Please read each of the following statements and select the response which most closely describes your level of agreement. As with all questionnaires in this study, your responses will be anonymous.

Closed-ended (forced response): Response Options:

Strongly Disagree	Disagree	Neither agree nor disagree	Agree	Strongly Agree
1	2	3	4	5

1. The Check was well presented
2. The items in the Flourishing Check were easy to understand
3. The items were relevant to early childhood flourishing
4. The Check covered all the areas that I believe are important to early childhood flourishing
5. The items were applicable to my child
6. The check was easy for me to complete
7. The questions were appropriate for the age of my child
8. I would use the Flourishing Check again (For example, for other children, if any, or the same child at another time)
9. The response options (Never, Rarely, Sometime, Often, Always) fit well with the questions
10. I felt comfortable answering each question

Appendix F: Key and Exploratory Acceptability and Feasibility Items for Evaluation of the Pathway Tool and Flourishing Check

Pathway Tool

Key acceptability items

Questionnaire items

- The Pathway Tool contains a good balance of online resources and face to face programs and/or services.
- The Pathway Tool contains a good amount of:
    d. Audio Resources
    e. Video Resources
    f. Written resources
- [If YES is selected for: Did you create a user account within the Pathway Tool? (Y/N) If you did not use any of these functions, please select the "NA" option]:
    c. The feedback from the Flourishing Check within the Pathway Tool was helpful.
    e. I found the ability to set up child profiles helpful.
    g. I found the ability to add additional family members helpful.

Acceptability items for exploration and modification

Questionnaire items

- The language in the Pathway Tool is easy for me to understand.
- The different pages in the Pathway Tool website are easy to navigate.
- The way resources are organised within the tool makes it easy to find what I need.
- The information in the Pathway Tool is presented in an order that is easy to understand.
- The visual cues (e.g., icons, keywords) in the Pathway Tool draw my attention to important information.
- The text on screen is easy to read.

Other themes that emerge from the focus groups

Website engagement metrics

Key feasibility items

Questionnaire items

- I understand the purpose of the Pathway Tool.
- The information in the Pathway Tool is relevant to me.
- The Pathway Tool includes a good amount of information on:
    a. Online resources
    b. Face to face programs and/or services
- The Pathway Tool helps me find resources and programs that are relevant to me or my child.
- I believe the Pathway Tool can help me support my child/children.
- I am likely to engage with services or programs I found through the Pathway Tool.
- I am likely to engage with online resources I found through the Pathway Tool.
- I am likely to use the Pathway Tool again in the future.
- Did you create a user account within the Pathway Tool? (Y/N) If you did not use any of these functions, please select the "NA" option.
    [IF YES]
    a. I was able to set up my user account easily.
    b. I was able to save resources easily.
    d. I was able to set up child profiles easily.
    f. I was able to add additional family members easily.

Feasibility items for exploration and modification

Questionnaire items

- The search filters make it easy to find what I need.
- I can identify at least one action I can take based on the Pathway Tool.

Other themes that emerge from the focus groups

Website engagement metrics

Flourishing Check

Key acceptability items

Questionnaire items

- The Check was well presented
- The items were relevant to early childhood flourishing
- The Check covered all the areas that I believe are important to early childhood flourishing
- The items were applicable to my child
- I would use the Flourishing Check again (For example, for other children, if any, or the same child at another time)
- I felt comfortable answering each question

Acceptability items for exploration and modification

Questionnaire items

- The response options (Never, Rarely, Sometime, Often, Always) fit well with the questions

Other themes that emerge from the focus groups

Key feasibility items

Questionnaire items

- The items in the Flourishing Check were easy to understand
- The check was easy for me to complete
- The questions were appropriate for the age of my child

Feasibility items for exploration and modification

Other themes that emerge from the focus groups

Note: this will mainly be covered by the psychometric analyses
